# Causal Associations of Obesity With Achilles Tendinopathy: A Two-Sample Mendelian Randomization Study

**DOI:** 10.3389/fendo.2022.902142

**Published:** 2022-06-14

**Authors:** Lijuan He, Tingting Yu, Wei Zhang, Baojian Wang, Yufeng Ma, Sen Li

**Affiliations:** ^1^ DongFang Hospital, Beijing University of Chinese Medicine, Beijing, China; ^2^ School of Life Sciences, Beijing University of Chinese Medicine, Beijing, China; ^3^ Beijing University of Chinese Medicine Third Affiliated Hospital, Beijing, China

**Keywords:** Achilles tendinopathy, body mass index, obesity, Mendelian randomization, causality

## Abstract

**Background:**

Achilles tendinopathy (AT) is associated with severe pain and is the cause of dysfunction and disability that are associated with significant reduction in social and economic benefits. Several potential risk factors have been proposed to be responsible for AT development; however, the results of observational epidemiological studies remain controversial, presumably because the designs of these studies are subject to residual confounding and reverse causality. Mendelian randomization (MR) can infer the causality between exposure and disease outcomes using genetic variants as instrumental variables, and identification of the causal risk factors for AT is beneficial for early intervention. Thus, we employed the MR strategy to evaluate the causal associations between previously reported risk factors (anthropometric parameters, lifestyle factors, blood biomarkers, and systemic diseases) and the risk of AT.

**Methods:**

Univariable MR was performed to screen for potential causal associations between the putative risk factors and AT. Bidirectional MR was used to infer reverse causality. Multivariable MR was conducted to investigate the body mass index (BMI)-independent causal effect of other obesity-related traits, such as the waist-hip ratio, on AT.

**Results:**

Univariable MR analyses with the inverse-variance weighted method indicated that the genetically predicted BMI was significantly associated with the risk of AT (*P*=2.0×10^-3^), and the odds ratios (95% confidence intervals) is 1.44 (1.14−1.81) per 1-SD increase in BMI. For the other tested risk factors, no causality with AT was identified using any of the MR methods. Bidirectional MR suggested that AT was not causally associated with BMI, and multivariable MR indicated that other anthropometric parameters included in this study were not likely to causally associate with the risk of AT after adjusting for BMI.

**Conclusions:**

The causal association between BMI and AT risk suggests that weight control is a promising strategy for preventing AT and alleviating the corresponding disease burden.

## Introduction

The Achilles tendon is the largest, most powerful tendon of the human body, which connects muscles, including gastrocnemius and soleus, to the heel bone (1). It plays an essential role in walking, standing, and maintaining body balance (1). Achilles tendinopathy (AT), also known as Achilles tendinitis, is a common disease in athletes whose Achilles tendon is subject to repeated and excessive stress (2, 3). For instance, the annual incidence rate of AT in competitive runners is approximately 8% and the lifetime incidence rate of AT is as high as 50% in elite distance runners (4). However, AT does not occur only in athletes, and nonathletes with sedentary behaviors constitute an important proportion of patients affected with AT (5). AT is associated with severe pain, and Achilles tendon becomes abnormal and increasingly fragile and fibrotic during AT, which represents a sign of its degeneration (2). Thus, AT is a normal cause of dysfunction and disability that significantly reduces social and economic benefits (6). Despite the issues associated with AT, the pathological mechanism underlying this condition remains largely unknown (7). Several factors have been proposed as contributors to the etiology of AT, including intrinsic (e.g., adiposity and high cholesterol) and extrinsic factors (e.g., physical activity-related factors) (8). However, results from conventional epidemiological studies remain controversial (9). For instance, one study showed a dose-response relationship between body mass index (BMI) and the risk of AT, whereas the case-control design of this study prevented the inference of causality (9). By contrast, the relationship between BMI/body weight and AT has been classified as “limited evidence for no association” in a systematic review investigating the clinical risk factors of AT (10).

Mendelian randomization (MR) can infer causality between exposure and disease outcomes by using genetic variants as instrumental variables (11). Genetic variants that are strongly associated with exposure should also be associated with outcome if causality exists between exposure and outcome (12). MR is based on the random assignment of genetic variation during meiosis, which makes MR studies less affected by residual confounding than conventional observational studies (13). Furthermore, MR studies are less subject to reverse causality because the genetic variants are fixed at birth and normally cannot be modulated by the outcomes (12).

In this study, univariable MR was performed to screen for potential causal association among ten putative risk factors (e.g., BMI) and AT. The causal effect of AT on BMI was studied using bidirectional MR. Multivariable MR was conducted to investigate the BMI-independent effect of other obesity-related traits, such as waist-hip ratio (WHR), on AT. To our knowledge, this is the first MR study to systematically infer the causality between clinical risk factors and AT.

## Methods

### Study Design

MR uses genetic variation, mostly single nucleotide polymorphisms (SNPs), as instrumental variables (IVs) to investigate the causality between traits and diseases. Valid instrumental variables need to fulfill three assumptions (14): (1) IVs are strongly associated with exposure; (2) IVs only affect the outcome through the exposure and have no direct association with the outcome; (3) IVs are independent of the confounders of the investigating association, i.e., the employed IVs should not have horizontal pleiotropy, as indicated by Assumptions (2) and (3). The two-sample design of MR refers to the use of two different study populations for the exposure and the outcome. In this study, we evaluated the causal associations of various potential clinical risk factors with AT by employing summary-level datasets generated in large genome-wide association studies (GWAS). The summary statistics of IVs were extracted from GWAS datasets of both exposure and outcome to perform the MR analyses. We first performed univariable MR analyses to screen for causality among ten putative clinical risk factors (e.g., BMI) and the risk of AT. Considering that individuals with AT are like to be physically inactive and have substantial weight gain, we next tested whether an increased risk of AT causally resulted in a higher BMI using bidirectional MR. To further investigate the potential causal relationship between other anthropometric parameters (e.g., WHR) and the risk of AT, multivariable MR analyses were conducted, in which BMI was adjusted.

### Data Sources

The SNPs for anthropometric measurements including BMI, waist circumference (WC), hip circumference (HC), WHR, whole-body fat mass (WBFM), whole-body fat-free mass (WBFFM), and whole-body fat percentage (WBF%) that reached a genome-wide significance level (*P*<5×10^–8^) were extracted from the GWAS datasets from the Genetic Investigation of ANthropometric Traits (GIANT) consortium and/or the UK Biobank (UKB) (15, 16). The SNPs obtained for each of these exposures were then clumped using a linkage disequilibrium (LD) parameter of R^2^>0.001 when they were within a physical distance of 10,000 kb. We also excluded SNPs that were palindromic and had an intermediate allele frequency, as previously described (17). The Axivity-AX3 triaxial accelerometer was used to measure the physical activity of the participants in the UKB for seven days, and the IVs selected for the accelerometer-based physical activity were the same as in the literature (18, 19). For smoking, the IVs used in our study were a genetic proxy of lifetime smoking behavior that integrates smoking duration, heaviness, and cessation (20), whereas the GWAS of drinking status was based on the participants’ self-reported number of drinks per week (21, 22). The IVs previously showing significant associations with systolic blood pressure at the genome-wide level in the dataset from the International Consortium for Blood Pressure (ICBP) were employed in our study (23). Genetic instruments for lipid traits, including LDL-C, TG, and HDL-C, were obtained from a GWAS dataset of 35 biomarkers from the UKB (24, 25). IVs of type 2 diabetes were obtained from a meta-analysis of datasets from the DIAbetes Genetics Replication and Meta-analysis (DIAGRAM), Genetic Epidemiology Research on Adult Health and Aging (GERA) cohort, and UKB as previously reported (12, 26). Genetically determined chronic kidney disease (CKD) was proxied using the SNPs from the dataset of the CKDGen Consortium (27, 28). The F-statistics of the IVs were calculated in accordance with a published method (29), and all exposures had mean F-statistics of over 10 (**Supplementary Table 1**). For outcome, the summary statistics of AT were from FinnGen R5 release, where AT was defined by the International Classification of Diseases (ICD)-10 code M76.6 or ICD-9 code 7267A. In our sensitivity analyses, a GWAS dataset with a study population of mixed ethnicity from the GERA cohort was employed, in which the outcome phenotype was Achilles tendon injury, including AT and Achilles tendon rupture (30). If the IVs for exposure were not available in the outcome dataset, proxy SNPs with high LD (R^2^>0.8) were used.

### Statistical Analysis

In the univariable MR, we inferred the potential causality between exposures and outcomes by using various methods based on different assumptions: inverse-variance weighted (IVW) method, MR-Egger method, weighted median method, and Mendelian Randomization Pleiotropy RESidual Sum and Outlier (MR-PRESSO). In the IVW method, the Wald ratio for each of the IVs will be meta-analysed to examine the causal association. The IVW method assumes that all included genetic variants are effective instrumental variables, which is different from the MR-Egger method, which remains functional if all IVs are invalid. Moreover, MR-Egger uses an intercept term to examine potential pleiotropy. The weighted median method is intermediate and requires that the valid variable must be at least 50%. MR-PRESSO can detect and exclude potential outliers in MR analyses, providing results with a pleiotropy correction. Heterogeneity was examined using the Cochrane Q value. The effect of each included IV on the causal association was addressed by the leave-one-out method, which removed each SNP individually and analyzes the estimate of the remaining SNPs by using IVW methods. Scatter plots were generated to visualize the MR results. Power estimation was performed using an online tool (31). In the bidirectional MR analyses, we relaxed the thresholds for including SNPs as IVs to *P*<1× 10^-5^ because no SNP reached a genome-wide significant association with AT (*P*<5×10^–8^). This strategy of relaxing the threshold has been employed in MR studies (32, 33). Multivariable MR with IVW analyses was performed to study the causal effect of other obesity-related traits on AT after adjusting for BMI. Odds ratios (ORs) and 95% confidence intervals (CIs) were applied to represent causality estimates of putative clinical risk factors and AT, and Bonferroni correction was used for statistical significance judgment in both univariable and multivariable MR analyses. All analyses were performed using the R packages TwoSampleMR (17), MendelianRandomization (34), and MR-PRESSO (35).

## Results

### Univariable Mendelian Randomization to Screen for the Causal Associations Between Putative Clinical Risk Factors and AT

**Figure 1:**
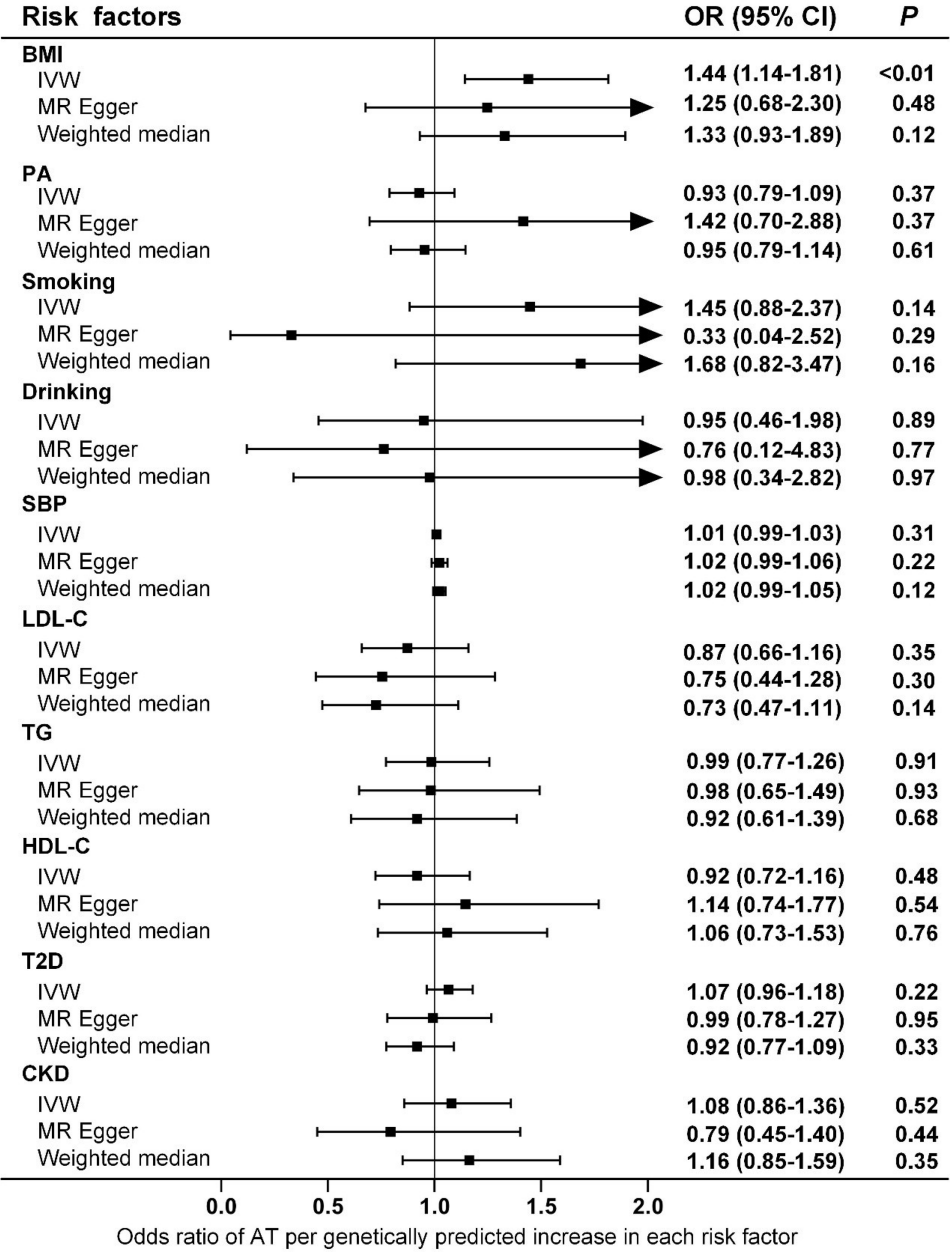
Associations of genetically predicted risk factors with risk of AT as examined by three MR methods. MR, Mendelian Randomization; IVW, inverse-variance weighted; BMI, body mass index; PA, physical activity; SBP, systolic blood pressure; LDL-C, low-density lipoprotein cholesterol; TG, triglyceride; HDL-C, high-density lipoprotein cholesterol; T2D, type 2 diabetes; CKD, chronic kidney disease; AT, Achilles tendinopathy; OR, Odds ratios; CI, confidence intervals.

**Figure 2:**
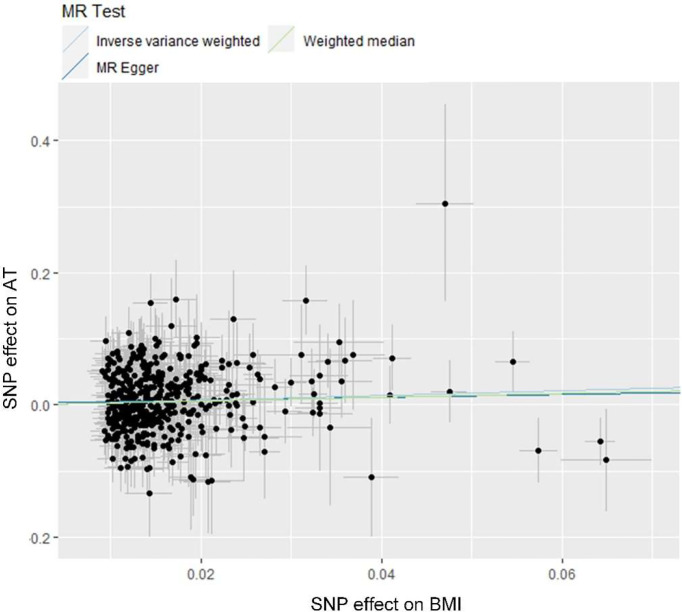
Scatter plot showing the causal effect of BMI on AT. MR, Mendelian Randomization; BMI, body mass index; AT, Achilles tendinopathy.

Ten clinical risk factors reported to be associated with AT in conventional epidemiological studies were included in the univariable MR analyses. Analyses using the IVW method indicated that the genetically predicted BMI was significantly associated with the risk of AT after Bonferroni correction for multiple comparison (*P*<5.0×10^-3^), and the OR (95% CIs) was 1.44 (1.14-1.81) for AT per 1-SD increase in BMI (**Figure 1** and **Figure 2**). No significant heterogeneity was found in this analysis (*P*=0.15) (**Supplementary Table 2**), and the leave-one-out analysis suggested that the results were not driven by a single SNP (**Supplementary Figure 1**). Furthermore, the directions of the association between BMI and AT risk were the same when using the MR-Egger and weighted median methods (**Figure 1**). As evaluated by the intercept term of the MR-Egger method, horizontal pleiotropy was nonsignificant (*P*=0.62) in the causality analysis between BMI and AT (**Supplementary Table 3**), which is also consistent with the results of MR-PRESSO in which no outlier IV was detected. The results of the power estimation suggested that the statistical power of this analysis was sufficient (>80%) (**Supplementary Table 4**). Next, we performed a sensitivity analysis in which the outcome was changed to Achilles tendon injury, including AT and Achilles tendon rupture (30), and the causal effect of BMI was consistently revealed (**Supplementary Table 5**). As another sensitivity analysis, we used a set of less IVs for BMI identified by Locke et al. (36), and the results also revealed a causal association between BMI and the risk of AT (**Supplementary Table 5**). For the other nine putative clinical risk factors, no causality with AT was identified using any of the MR methods (**Figure 1** and **Supplementary Figure 2**).

### Bidirectional Mendelian Randomization to Test the Causal Effect of AT on BMI

Twelve SNPs associated with AT at a relaxed threshold (*P*<1×10^–5^) were selected as IVs for AT in the bidirectional MR. A significant level of heterogeneity was detected in the analyses (**Supplementary Table 2**), and the results of MR with the multiplicative random-effect IVW method indicated that AT was not causally associated with BMI (**Supplementary Table 5**). Similar results were obtained using MR-PRESSO, which excluded outlier SNPs (**Supplementary Table 6**). We also performed a sensitivity analysis using Achilles tendon injury as exposure, which consistently indicated that this phenotype did not cause an increase in BMI (**Supplementary Table 5**).

### Multivariable Mendelian Randomization to Examine the BMI-Adjusted Causal Association of Other Anthropometric Traits With AT

**Figure 3:**
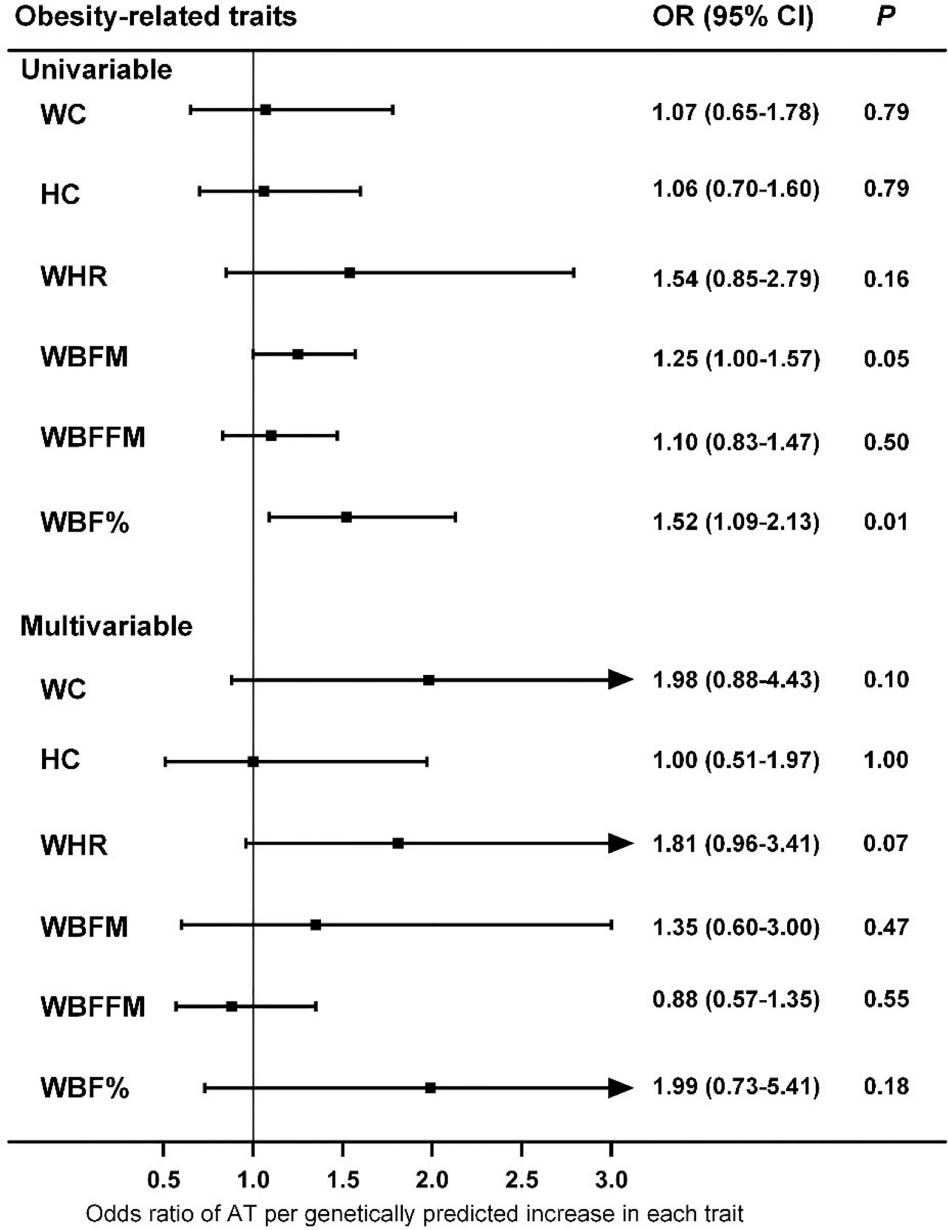
Associations of genetically predicted obesity-related traits with risk of AT as examined by univariable and multivariable MR with the IVW method. MR, Mendelian Randomization; IVW, inverse-variance weighted; WC, waist circumference; HC, hip circumference; WHR, waist-hip ratio; WBFM, whole body fat mass; WBFFM, whole body fat-free mass; WBF%, whole body fat percentage; AT, Achilles tendinopathy; OR, Odds ratios; CI, confidence intervals.

To evaluate the causal effect of other obesity-related traits on AT, we first performed univariable MR. The results suggested that only WBF% was causally associated with the risk of AT at the nominal significance level (*P*=0.01) (**Figure 3**), which did not survive the Bonferroni correction (*P*_threshold_=0.05/6). Because obesity-related traits are highly related to BMI, we also performed multivariable MR analysis, and the results indicated that none of these traits were causally associated with the risk of AT after adjusting for BMI (**Figure 3**).

## Discussion

In this study, we tested the potential causal association between ten putative AT risk factors and the risk of AT by using a two-sample MR design and identified the causality between high BMI and increased risk of AT. Bidirectional MR revealed that an elevated risk of AT was not causally associated with increased BMI. Furthermore, multivariable MR analysis indicated that other anthropometric parameters, such as WHR, were not likely to be associated with the risk of AT.

Obesity is a global epidemic, and our results suggest that weight control is effective and necessary for the prevention and control of AT. Compared with intrinsic factors such as age and sex, overweight and obesity are more easily to control and therefore can serve as promising targets for AT if they casually lead to an increased risk of AT development and/or progression. However, conventional epidemiological studies have presented inconclusive evidence of an association between BMI and AT risk (9). One study indicated that in addition to overuse, individuals who were overweight and less physically active were also prone to tendinopathy (37). In a study population in the United States, the BMI of patients with Achilles pathology was significantly higher than that of patients without Achilles pathology, independent of age (38). A report from Norway showed that military conscripts with higher BMIs were more likely to have musculoskeletal injuries such as AT (39). Moreover, overweight or obesity increased the risk of AT by 2.6 to 6.6 times (9), and a significant association between obesity and AT was observed in both male and female participants (40). Other studies have found that the incidence of AT is not necessarily associated with BMI. In a study population of male officer cadets, BMI was not significantly different among subjects with and without Achilles tendon overuse injury (41). Furthermore, a systematic review indicated no association between BMI/body weight and AT, although the evidence from the literature was limited (10). To our knowledge, the current investigation is the first study to infer causality between BMI and AT, and both the main analysis and sensitivity analyses consistently support the hypothesis that BMI is causally associated with AT, with an OR (95% CIs) of 1.44 (1.14-1.81), but the reverse is not true (**Figure 1** and **Supplementary Table 5**). Notably, the magnitude of estimates from MR analyses is normally larger than that of observational studies because the BMI proxied by genetic instruments in MR studies is a measurement of lifelong exposure, and the BMI measured in conventional epidemiological studies usually reflects short-term exposure (42). Moreover, the results of the MR study should be interpreted carefully because it is appropriate to interpret such results as statistical test results for causal relationships but not the expected effects of clinical interventions at a particular time point (43). For other anthropometric parameters, conventional epidemiological studies have suggested an increased prevalence of AT in participants with a central fat distribution (44). However, our MR analyses suggested that WHR, as a measure of body fat distribution, did not increase the risk of AT (**Figure 3**).

Tendons are mechanically sensitive tissues that respond to mechanical loads of varying magnitudes, frequencies, and durations (45). However, if the tendon is overloaded, its muscle strength and elasticity may become unbalanced, irritating the tendon and causing tendinopathy (2). Considering that eight times the body weight can be placed on the tendon during running, a slight increase in body weight results in a significant increase in tendon loading (46). Thus, overweight is likely to cause tendon loading beyond the threshold for normal physiological function of the tendon and promote its degeneration (44). Indeed, tendon structural alterations can be revealed by ultrasound in overweight subjects (44). Adipose tissue in obese individuals produces bioactive peptides, such as adipokines, which modulate the function of tendon cells (e.g., tenocytes) and alter the tendon structure by inducing nitric oxide synthase and regulating the production of metalloproteinases that contribute to the increased level of degradation products (47–49). Moreover, excessive generation of proteoglycans by tenocytes contributes to water retention and tendon swelling (50). Obesity is also a known contributor to the development of insulin resistance, which promotes subclinical glucose intolerance and accumulation of glycotoxins that can cross-link with the collagen fibers of the tendon (51). In addition, individuals with obesity and impaired insulin sensitivity have higher levels of prostaglandin E2, indicating a state of chronic low-grade inflammation, which may interfere with tendon healing (44). More specifically, increased migration of immune cells into adipose tissue has been found in obese subjects, leading to a reduced level of circulating transforming growth factor-beta (TGF-β), a profibrotic factor, which makes it difficult to heal the Achilles tendon (52, 53). If the Achilles tendon fails to heal continuously, prolonged fibrogenesis occurs, thereby inducing the deposition of the extracellular matrix and impairing tendon function (44).

In addition to obesity, epidemiological evidence suggests the presence of other potential clinical risk factors for AT. For example, an elevated risk of AT has been found in the United States military personnel with moderate alcohol use (54). Acute ruptures of the Achilles tendon are also associated with hypertension, whose complications may contribute to the reduced vascularity and the healing potential of the body (40). Systemic factors (e.g., circulating lipids) may reduce the ability of the tendon to handle loads and thus lead to AT (55). Indeed, patients with Achilles tendon rupture have higher concentrations of cholesterol (56), and biopsies from patients with AT exhibit increased levels of the esterified fraction of cholesterol (57). In addition, a higher incidence of diabetes has been reported in individuals with Achilles tendon ruptures, suggesting its involvement in the pathogenesis of AT (58). Renal dysfunction has also been proposed as an independent risk factor for AT in patients who have received a heart transplant (59). However, none of the potential clinical risk factors reported in conventional epidemiological studies were causally associated with AT in our MR analyses (**Figure 1**). Interestingly, although AT is generally described as “overuse injury”, a systemic review indicates that both the level and performance of physical activity are not associated with AT, presumably because sudden load changes are more important to the pathogenesis of AT than the total amount of load that has been used as an indicator of physical activity in the majority of the studies (10). This hypothesis is supported by our MR analyses that did not reveal any causality between genetically determined physical activity and the risk of AT.

This study has several strengths. First, the MR design reduced biases introduced by residual confounding and reverse causality, which may lead to false-positive results in conventional observational studies. Second, the two-sample MR analysis using summary statistics from independent, large GWAS for exposures and outcomes separately improved the statistical power to examine their causal association. Third, the use of multiple SNPs as IVs for each of the putative clinical risk factors of AT enabled us to analyze potential directional pleiotropy. Furthermore, we employed numerous methods, such as MR-Egger, multivariable MR, and MR-PRESSO, for sensitivity analyses to detect and adjust for pleiotropy. Fourth, the bias introduced by population stratification was reduced in our analyses because the majority of the participants in the original GWAS were European. This study also has several limitations. First, compared with other diseases (e.g., cardiovascular diseases) studied in MR, the number of AT cases used in the production of the GWAS summary statistics was relatively small, leading to a reduced statistical power to reveal the causal association with the exposures included in this study. Second, although we employed multiple MR methods to deal with the potential directional pleiotropy, as a theoretical weakness of the MR studies, the remaining pleiotropy could not be completely ruled out.

## Conclusion

This comprehensive MR study has revealed a causal association between BMI and the risk of AT, suggesting that weight control is a promising strategy for preventing AT and alleviating the corresponding disease burden.

## Data Availability Statement

The original contributions presented in the study are included in the article/**Supplementary Material**. Further inquiries can be directed to the corresponding authors.

## Ethics Statement

The GWAS included in this work were approved by their relevant review board, and informed consent were given by all participants.

## Author Contributions

SL and YM designed the study. LH and TY performed the statistical analyses, and drafted the manuscript. WZ and BW critically reviewed the manuscript. All authors contributed to the article and approved the submitted version.

## Funding

This study is supported by the Capital’s Funds for Health Improvement and Research (Grant No. 2020-2-7036).

## Conflict of Interest

The authors declare that the research was conducted in the absence of any commercial or financial relationships that could be construed as a potential conflict of interest.

## Publisher’s Note

All claims expressed in this article are solely those of the authors and do not necessarily represent those of their affiliated organizations, or those of the publisher, the editors and the reviewers. Any product that may be evaluated in this article, or claim that may be made by its manufacturer, is not guaranteed or endorsed by the publisher.

## Abbreviations

SNP, single nucleotide polymorphism; GWAS, genome-wide association studies; MR, Mendelian Randomization; IVW, inverse-variance weighted; MR-PRESSO, Mendelian Randomization Pleiotropy RESidual Sum and Outlier; ICD, international classification of diseases; LD, linkage disequilibrium; TGF-β, transforming growth factor-beta; BMI, body mass index; WC, waist circumference; HC, hip circumference; WHR, waist-hip ratio; WBFM, whole body fat mass; WBFFM, whole body fat-free mass; WBF%, whole body fat percentage; PA, physical activity; SBP, systolic blood pressure; LDL-C, low-density lipoprotein cholesterol; TG, triglyceride; HDL-C, high-density lipoprotein cholesterol; T2D, type 2 diabetes; CKD, chronic kidney disease; AT, Achilles tendinopathy; UKB, UK Biobank; ICBP, International Consortium for Blood Pressure; GIANT, Genetic Investigation of ANthropometric Traits; DIAGRAM, DIAbetes Genetics Replication and Meta-analysis; GERA, Genetic Epidemiology Research on Adult Health and Aging; IVs, Instrumental variable.
