# Supplementary material for: Causal Associations of Obesity With Achilles Tendinopathy: A Two-Sample Mendelian Randomization Study
Source: Front Endocrinol (Lausanne). 2022 Jun 14;13:902142. doi: 10.3389/fendo.2022.902142 (PMC9238354; doi:10.3389/fendo.2022.902142)
Supplement: Supplementary file 1 [file DataSheet_1.pdf]

Supplementary Table 1. The F-Statistic of SNPs used as IVs.

Variables	Sample Sizes	Cases	Number of SNPs used as IVs	F-Statistic
BMI	681275	NA	481	73.62
PA	91084	NA	8	33.75
Smoking	462690	NA	118	48.63
Drinking	941280	NA	39	33.06
SBP	299024	NA	255	27.85
LDL-C	318340	NA	147	98.13
TG	318674	NA	129	115.32
HDL-C	291830	NA	136	109.67
T2D	659316	62892	133	71.83
CKD	480698	41395	19	64.73
WC	232101	NA	40	50.09
HC	213038	NA	51	55.43
WHR	212244	NA	28	45.33
WBFM	454137	NA	412	62.26
WBFFM	454850	NA	523	85.32
WBF%	331117	NA	245	52.58
BMI (Locke et al.)	322154	NA	95	49.98
AT	166307	1625	12	21.80
Achilles tendon injury (tendinopathy, rupture or repair)	102979	5148	13	21.18

Abbreviation: SNP: single nucleotide polymorphism; BMI: body mass index; WC: waist circumference; HC: hip circumference; WHR: waist-hip ratio; WBFM: whole body fat mass; WBFFM: whole body fat-free mass; WBF%: whole body fat percentage; PA: physical activity; SBP: systolic blood pressure; LDL-C: low-density lipoprotein cholesterol; TG: triglyceride; HDL-C: high-density lipoprotein cholesterol; T2D: type 2 diabetes; CKD: chronic kidney disease; AT: Achilles tendinopathy; UKB: UK Biobank; ICBP: International Consortium for Blood Pressure; GIANT: Genetic Investigation of ANthropometric Traits; DIAGRAM: DIAbetes Genetics Replication and Meta-analysis; GERA: Genetic Epidemiology Research on Adult Health and Aging; NA: not available; IVs: Instrumental variable.

Supplementary Table 2. Results of heterogeneity tests.

Exposure	Outcome	Method	Q	P
BMI	AT	MR Egger	511.54	0.147
BMI	AT	Inverse variance weighted	511.81	0.152
PA	AT	MR Egger	9.28	0.158
PA	AT	Inverse variance weighted	11.49	0.119
Smoking	AT	MR Egger	114.26	0.528
Smoking	AT	Inverse variance weighted	116.42	0.498
Drinking	AT	MR Egger	33.73	0.623
Drinking	AT	Inverse variance weighted	33.80	0.664
SBP	AT	MR Egger	229.17	0.856
SBP	AT	Inverse variance weighted	229.87	0.859
LDL-C	AT	MR Egger	182.11	0.020
LDL-C	AT	Inverse variance weighted	182.62	0.021
TG	AT	MR Egger	131.51	0.374
TG	AT	Inverse variance weighted	131.51	0.398
HDL-C	AT	MR Egger	121.50	0.773
HDL-C	AT	Inverse variance weighted	122.91	0.764
T2D	AT	MR Egger	131.82	0.463
T2D	AT	Inverse variance weighted	132.21	0.478
CKD	AT	MR Egger	20.41	0.254
CKD	AT	Inverse variance weighted	22.01	0.232
WC	AT	MR Egger	47.79	0.133
WC	AT	Inverse variance weighted	47.79	0.158
HC	AT	MR Egger	61.02	0.116
HC	AT	Inverse variance weighted	62.76	0.106
WHR	AT	MR Egger	24.79	0.531
WHR	AT	Inverse variance weighted	28.54	0.383
WBFM	AT	MR Egger	403.25	0.585
WBFM	AT	Inverse variance weighted	403.25	0.598
WBFFM	AT	MR Egger	575.89	0.048
WBFFM	AT	Inverse variance weighted	575.90	0.051
WBF%	AT	MR Egger	222.89	0.818
WBF%	AT	Inverse variance weighted	222.92	0.830
BMI (Locke et al.)	AT	MR Egger	98.77	0.322
BMI (Locke et al.)	AT	Inverse variance weighted	99.01	0.342
BMI	Achilles tendon injury (tendinopathy, rupture or repair)	MR Egger	504.97	0.190
BMI	Achilles tendon injury (tendinopathy, rupture or repair)	Inverse variance weighted	505.15	0.197
AT	BMI	MR Egger	69.46	<0.001
AT	BMI	Inverse variance weighted	74.89	<0.001
Achilles tendon injury (tendinopathy, rupture or repair)	BMI	MR Egger	24.79	0.010
Achilles tendon injury (tendinopathy, rupture or repair)	BMI	Inverse variance weighted	24.79	0.016

Abbreviation: BMI: body mass index; WC: waist circumference; HC: hip circumference; WHR: waist-hip ratio; WBFM: whole body fat mass; WBFFM: whole body fat-free mass; WBF%: whole body fat percentage; PA: physical activity; SBP: systolic blood pressure; LDL-C: low-density lipoprotein cholesterol; TG: triglyceride; HDL-C: high-density lipoprotein cholesterol; T2D: type 2 diabetes; CKD: chronic kidney disease; AT: Achilles tendinopathy.

Supplementary Table 3. Results of MR-Egger pleiotropy tests.

Exposures	Outcomes	Intercept	SE	P
BMI	AT	0.0025	0.0051	0.619
PA	AT	-0.1105	0.0925	0.277
Smoking	AT	0.0228	0.0155	0.145
Drinking	AT	0.0051	0.0202	0.801
SBP	AT	-0.0044	0.0053	0.405
LDL-C	AT	0.0049	0.0077	0.523
TG	AT	0.0001	0.0068	0.984
HDL-C	AT	-0.0082	0.0069	0.237
T2D	AT	0.0054	0.0086	0.532
CKD	AT	0.0284	0.0246	0.264
WC	AT	-0.0007	0.0246	0.977
HC	AT	0.0223	0.0188	0.242
WHR	AT	0.0657	0.0339	0.064
WBFM	AT	0.0002	0.0057	0.979
WBFFM	AT	0.0005	0.0046	0.908
WBF%	AT	0.0017	0.0095	0.857
BMI (Locke et al.)	AT	-0.0061	0.0126	0.631
BMI	Achilles tendon injury (tendinopathy, rupture or repair)	-0.0013	0.0032	0.684
AT	BMI	0.0049	0.0055	0.398
Achilles tendon injury (tendinopathy, rupture or repair)	BMI	-0.0001	0.0033	0.987

Abbreviation: BMI: body mass index; WC: waist circumference; HC: hip circumference; WHR: waist-hip ratio; WBFM: whole body fat mass; WBFFM: whole body fat-free mass; WBF%: whole body fat percentage; PA: physical activity; SBP: systolic blood pressure; LDL-C: low-density lipoprotein cholesterol; TG: triglyceride; HDL-C: high-density lipoprotein cholesterol; T2D: type 2 diabetes; CKD: chronic kidney disease; AT: Achilles tendinopathy; SE: standard error.

Supplementary Table 4. Results of power estimation.

Exposures	Outcome	Cases	Controls	Sample size	OR with 80% power
BMI	AT	1625	164682	166307	<0.68 or >1.32
PA	AT	1625	164682	166307	>2.28
Smoking	AT	1625	164682	166307	<0.38 or >1.63
Drinking	AT	1625	164682	166307	<0.13 or >1.89
SBP	AT	1625	164682	166307	<0.55 or >1.45
LDL-C	AT	1625	164682	166307	<0.68 or >1.33
TG	AT	1625	164682	166307	<0.68 or >1.33
HDL-C	AT	1625	164682	166307	<0.69 or >1.31
T2D	AT	1625	164682	166307	<0.45 or >1.55
CKD	AT	1625	164682	166307	>2.29
WC	AT	1625	164682	166307	<0.27 or >1.75
HC	AT	1625	164682	166307	<0.41 or >1.6
WHR	AT	1625	164682	166307	<0.13 or >1.89
WBFM	AT	1625	164682	166307	<0.71 or >1.29
WBFFM	AT	1625	164682	166307	<0.78 or >1.22
WBF%	AT	1625	164682	166307	<0.65 or >1.35

Abbreviation: BMI: body mass index; WC: waist circumference; HC: hip circumference; WHR: waist-hip ratio; WBFM: whole body fat mass; WBFFM: whole body fat-free mass; WBF%: whole body fat percentage; PA: physical activity; SBP: systolic blood pressure; LDL-C: low-density lipoprotein cholesterol; TG: triglyceride; HDL-C: high-density lipoprotein cholesterol; T2D: type 2 diabetes; CKD: chronic kidney disease; AT: Achilles tendinopathy.

Supplementary Table 5. Results of MR analyses.

Exposures	Outcomes	Method	Number of SNP	Beta	SE	P
BMI (Locke et al.)	AT	MR Egger	95	0.680	0.507	0.183
BMI (Locke et al.)	AT	Inverse variance weighted	95	0.450	0.173	0.009
BMI (Locke et al.)	AT	Weighted median	95	0.470	0.262	0.073
BMI	Achilles tendon injury (tendinopathy, rupture or repair)	MR Egger	480	0.361	0.192	0.061
BMI	Achilles tendon injury (tendinopathy, rupture or repair)	Inverse variance weighted	480	0.289	0.074	<0.001
BMI	Achilles tendon injury (tendinopathy, rupture or repair)	Weighted median	480	0.213	0.125	0.087
AT	BMI	MR Egger	12	-0.011	0.023	0.636
AT	BMI	Inverse variance weighted	12	0.008	0.007	0.269
AT	BMI	Weighted median	12	0.002	0.005	0.671
Achilles tendon injury (tendinopathy, rupture or repair)	BMI	MR Egger	13	-0.001	0.025	0.962
Achilles tendon injury (tendinopathy, rupture or repair)	BMI	Inverse variance weighted	13	-0.002	0.008	0.835
Achilles tendon injury (tendinopathy, rupture or repair)	BMI	Weighted median	13	0.002	0.008	0.800

Abbreviation: SNP: single nucleotide polymorphism; BMI: body mass index; WC: waist circumference; HC: hip circumference; WHR: waist-hip ratio; WBFM: whole body fat mass; WBFFM: whole body fat-free mass; WBF%: whole body fat percentage; PA: physical activity; SBP: systolic blood pressure; LDL-C: low-density lipoprotein cholesterol; TG: triglyceride; HDL-C: high-density lipoprotein cholesterol; T2D: type 2 diabetes; CKD: chronic kidney disease; AT: Achilles tendinopathy; SE: standard error.

Supplementary Table 6. MR-PRESSO analyses between exposures and outcomes with outlier

Exposures	Outcomes	MR Analysis	Causal Estimate	Sd	P	P (MR.PRESSO.Global.Test)
LDL-C	AT	Raw	-0.135	0.144	0.350	0.020
		Outlier-corrected	-0.163	0.138	0.239	
AT	BMI	Raw	0.008	0.007	0.293	<0.001
		Outlier-corrected	0.006	0.004	0.151	
Achilles tendon injury (tendinopathy, rupture or repair)	BMI	Raw	-0.002	0.008	0.838	0.014
		Outlier-corrected	-0.006	0.006	0.350	

Abbreviation: BMI: body mass index; LDL-C: low-density lipoprotein cholesterol; AT: Achilles tendinopathy.

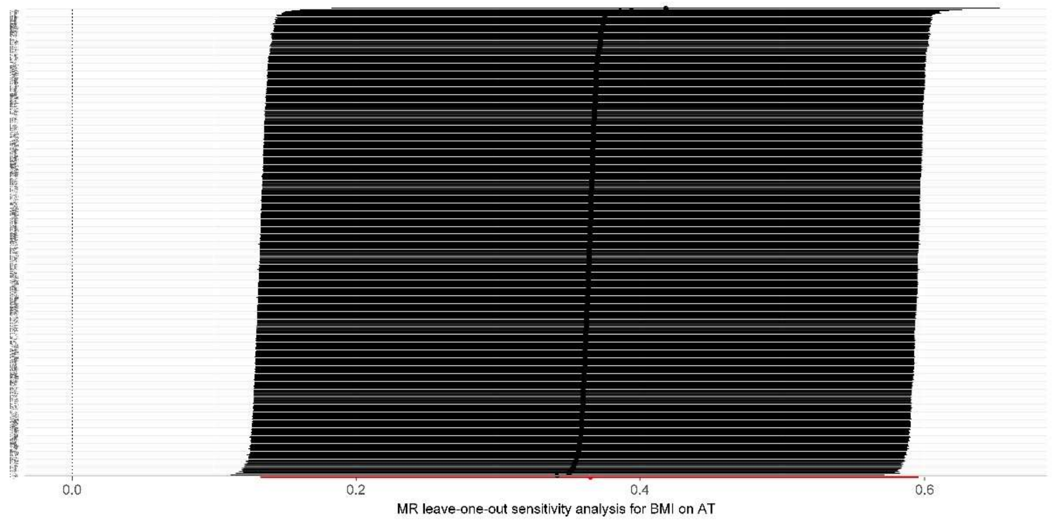

Supplementary Figure 1. Leave-one-out sensitivity analysis for BMI on AT. Abbreviation: MR: Mendelian Randomization; BMI: body mass index; AT: Achilles tendinopathy.

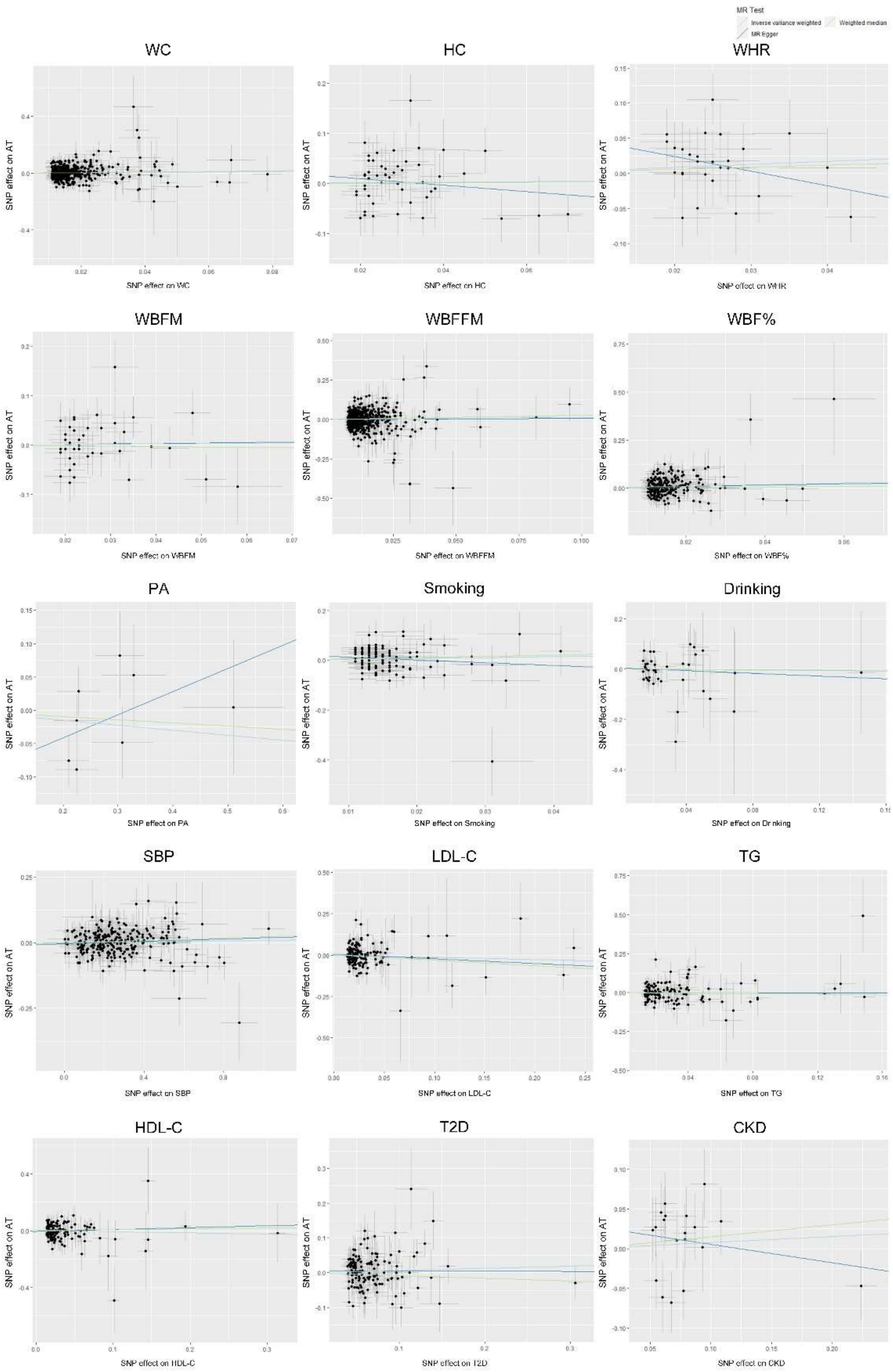

Supplementary Figure 2. Scatter plots showing effects of clinical risk factors on AT. Abbreviation: MR: Mendelian Randomization; WC: waist circumference; HC: hip circumference; WHR: waist-hip ratio; WBFM: whole body fat mass; WBFFM: whole body fat-free mass; WBF%: whole body fat percentage; PA: physical activity; SBP: systolic blood pressure; LDL-C: low-density lipoprotein cholesterol; TG: triglyceride; HDL-C: high-density lipoprotein cholesterol; T2D: type 2 diabetes; CKD: chronic kidney disease; AT: Achilles tendinopathy.
